# Optimizing Cellular Networks Enabled with Renewal Energy via Strategic Learning

**DOI:** 10.1371/journal.pone.0132997

**Published:** 2015-07-13

**Authors:** Insoo Sohn, Huaping Liu, Nirwan Ansari

**Affiliations:** 1 Division of Electronics & Electrical Engineering, Dongguk University—Seoul, Seoul, Republic of Korea; 2 School of Electrical Engineering and Computer Science, Oregon State University, Corvallis, Oregon, United States of America; 3 Department of Electrical and Computer Engineering, New Jersey Institute of Technology, Newark, New Jersey, United States of America; Tianjin University of Technology, CHINA

## Abstract

An important issue in the cellular industry is the rising energy cost and carbon footprint due to the rapid expansion of the cellular infrastructure. Greening cellular networks has thus attracted attention. Among the promising green cellular network techniques, the renewable energy-powered cellular network has drawn increasing attention as a critical element towards reducing carbon emissions due to massive energy consumption in the base stations deployed in cellular networks. Game theory is a branch of mathematics that is used to evaluate and optimize systems with multiple players with conflicting objectives and has been successfully used to solve various problems in cellular networks. In this paper, we model the green energy utilization and power consumption optimization problem of a green cellular network as a pilot power selection strategic game and propose a novel distributed algorithm based on a strategic learning method. The simulation results indicate that the proposed algorithm achieves correlated equilibrium of the pilot power selection game, resulting in optimum green energy utilization and power consumption reduction.

## Introduction

With the rapid increase in the world’s energy demand, the reduction of greenhouse gas (GHG) emissions to combat global warming has become one of the most important issues in today’s society. The information and communications technology (ICT) sector is a major energy consumption source, producing 2% of the global GHG [1], and the amount of contribution is increasing every year. Within the ICT sector, cellular networks are responsible for a large portion of the GHG emissions, and 70% of the cellular networks’ energy consumption is due to the base stations (BSs) [2]. Research work has been dedicated to advancing the green communication network technology, including self-healing networks [3], aimed at maintaining high levels of power saving performance based on complex network theory [4–6]. Other key green network techniques, with the objective of GHG minimization, are energy-efficient hardware design, cross-layer adaptive resource allocation, intelligent interference control, energy-aware cooperative BS, heterogeneous network deployment, and renewable energy enabled BS [7, 8]. Renewable energy is defined to include wind, solar, geothermal, biomass, and hydropower and is recognized as a critical element of a GHG energy economy [9]. In 2012, renewable energy provided an estimated 19% of global energy consumption, and in 2013, renewables accounted for more than 56% of the net additions to the global power capacity [10]. Based on this trend, renewable energy-powered cellular networks have been recognized as an important element among key green network techniques [11–13]. In [8], GHG emission savings and the cost of various heterogeneous network topologies using renewable energy sources were evaluated. In [10], the design and optimization issues of green energy enabled mobile networks were investigated.

Due to the significant variation in the quantity of green energy harvested and the limitations of energy storage, it is expected that future green BSs will be powered by multiple energy sources, including conventional on-grid energy. The fundamental design criterion in developing hybrid energy-based green BSs is the optimal utilization of green energy over a period of time based on energy arrival and energy consumption. In [14], the authors proposed the Intelligent Cell brEathing (ICE) algorithm that maximizes the utilization of green energy through adaptive cell breathing. ICE balances the users among BSs through cell breathing to minimize the maximal energy depleting rate, resulting in an increased number of users with green energy usage. However, ICE maximizes the green energy utilization at the expense of the overall energy efficiency.

Game theory is a branch of mathematics that is used to evaluate and optimize systems with multiple players with conflicting objectives [15], and it has been applied to solve various problems in wireless networks [16–20]. The regret matching procedure is an adaptive strategic learning method and has been applied to many problems based on game theory [21–23]. The key idea of the regret matching procedure is to adjust the players’ strategies based on regret measures, which are calculated using past observations. An important motivation for considering the regret matching procedure to solve a problem modeled based on game theory is that it guarantees convergence to a correlated equilibrium solution [24, 25]. In this paper, we formulate the green energy utilization and power consumption optimization problem as a strategic game and propose a simple distributed algorithm that solves the strategic game based on the regret matching procedure to enhance energy efficiency. The proposed algorithm achieves an equilibrium solution to the pilot power selection game, resulting in optimum green energy utilization and power consumption reduction.

The rest of the paper is organized as follows. In Section 2, we describe the green network model and ICE scheme, and the dynamic pilot power selection problem is formulated as a game theory problem. In Section 3, we propose a pilot power selection algorithm based on the regret matching procedure for green networks with convergence and comparative analysis. The numerical results of the proposed algorithm are given in Section 4, and conclusions are presented in Section 5.

## System Model

### Green Network Model

We consider a green cellular network consisting of *M* BSs and *I* users. The BSs are assumed to be powered by either on-grid energy or green energy. If the stored green energy in a BS is greater than the required power consumption to satisfy the user traffic demand, the BS will be powered by green energy. Otherwise, on-grid energy will be used to power the BS. We further assume that the BSs can adaptively adjust the cell coverage area by changing the pilot signal strength [25]. The cell coverage area is increased to attract mobile traffic and is decreased to offload mobile traffic to neighboring cells to maximize green energy usage and minimize system power consumption. The BSs update the cell coverage area every τ seconds by selecting an optimum pilot signal power from

$$P^{pl}=\left[p_{1}^{pl}\;p_{2}^{pl}\;\cdots \;p_{k}^{pl}\;\cdots \;p_{K}^{pl}\right],$$ (1)

where *K* is the maximum power level. To analyze the efficiency of the green energy utilization, we adopt the energy depleting rate (EDR) [11] as the metric for BS *m*, given below

$$R_{m}=\frac{\left(n_{m}P_{m}^{tx}+P_{m}^{s}\right)\tau }{E_{m}^{g}}\;,$$ (2)

where *R*_*m*_ is the EDR, *n*_*m*_ is the number of users associated with BS *m*, $P_{m}^{tx}$ is the transmit power, $P_{m}^{s}$ is the static power consumption, and $E_{m}^{g}$ is the current allocated green energy in BS *m*. Furthermore, the system power takes into account the BS energy consumption, including the green energy and on-grid energy, as defined below:

$$P^{T}=\sum_{m=1}^{M}E_{m}^{T}\;,$$ (3)

where *M* is the total number of BSs and $E_{m}^{T}$ denotes the total energy consumption in BS *m* defined as

$$E_{m}^{T}=\left\{ \begin{array}{l} n_{m}P_{m}^{tx}+P_{m}^{s}\;\text{if}\;R_{m}\geq 1\\ E_{m}^{g}\;\text{if}\;R_{m}<1 \end{array}\right.\;.$$ (4)

Here, it is assumed that a cell is always in the active state, i.e., there is at least one user requesting a service.

### ICE Scheme

In the ICE algorithm, the green energy utilization is optimized by adaptively adjusting the cell coverage area. The key metric used for the optimization process is the EDR, which is a normalized rate of energy consumption over the allocated green energy for a BS. ICE starts with the maximum pilot power level assignments and then reduces the pilot power of BSs with large EDR accordingly until the optimal solution is reached. ICE can be summarized as follows [11].

1. Initialize the pilot power of all the BSs in the system with the maximum pilot power level *K*.
2. Search for BSs with *EDR* > δ, where δ is the maximum EDR value at the current stage.
3. Reduce the pilot power accordingly as follows:

$$p_{h}^{\prime }=p_{h}-w_{h},$$ (5)

where $p_{h}^{\prime }$ is the new pilot power level, *h* is the BS index with *EDR* > δ, and *w*_*h*_ is an optimum pilot power decrement weight that guarantees EDR reduction.

4) The User-BS association state is updated based on the pilot power adjustments.
5) Pilot power optimization process steps 2, 3, and 4 are repeated until the following conditions are satisfied.
  - All of the BSs are included in the energy dependent set (EDS), where EDS is defined to be a set of BSs with *EDR* > δ.
  - All of the current pilot power levels of all of the BSs in the system are less than the optimum pilot power decrement weight.

## Game Problem Formulation

To maximize the green energy utilization and minimize the total energy consumption based on the green network model described above, the BSs are designed to optimize the cell coverage area through dynamic pilot power selection. We propose to model the pilot power selection problem as a strategic game such that 1) BSs are rewarded for choosing strategies that increase green energy utilization and 2) BSs are penalized for choosing strategies that increase system power consumption. The pilot power selection problem is formulated as the following strategic game

$$\Gamma =\langle M,{\left\{S_{m}\right\}}_{m\in M},{\left\{u_{m}\right\}}_{m\in M}\;\rangle \;.$$ (6)

Below are the components of the strategic game:

1. Set of players: *M* is the set of players corresponding to the BSs with the goal of maximizing its utility.
2. Set of strategies: *S*_*m*_ is the set of strategies for player *m* corresponding to the candidate power levels to be selected from $P^{pl}=\left[p_{1}^{pl}\;\cdots \;p_{K}^{pl}\right]$.
3. Utility function: *u*_*m*_ is the utility function defined as

$$u_{m}(s_{m},s_{-m})=R_{m}^{-1}(s_{m},s_{-m})-\alpha \;P^{T}(s_{m},s_{-m}),$$ (7)

where *s*_−*i*_ = [*s*_1_, ⋯, *s*_*i*−1_, *s*_*i*+1_, ⋯, *s*_*N*_], $R_{m}^{-1}$ is the inverse of the EDR metric representing the user green energy benefit, *P* ^*T*^ is the system power consumption that represents the cost of energy efficiency, and α is the weight factor for balancing the green energy benefit and the system power consumption cost.

The utility function is an important performance metric that measures the maximum green energy efficiency that can be achieved by BS *m* with the cost of system power consumption when pilot power level *k* is selected.

Furthermore, the best response strategy for BS *m* is given by the solution to the following maximization problem

$$\begin{array}{l} \underset{s_{m}\in S_{m}}{\text{max}}u_{m}(s_{m},s_{-m})\;,\\ \text{subject to}:\;p^{r}\;k\;d_{m,i}^{2}\leq P_{m}^{tx} \end{array}$$ (8)

where *p* ^*r*^ is the minimum reception power to satisfy the user QoS requirement, *k* is the constant path loss factor, d_m,i_ is the distance between the BS *m* and user *i*, and $P_{m}^{tx}$ is the transmit power. The player *m*’s utility function also depends on the strategy selection of all other players s_-m_ because the number of users and the current pilot power status of other cells are important factors in determining the optimum strategy for player *m*.

The equilibrium of a strategic game is defined to be a combination of strategies containing the best strategy for all players, and we adopt the correlated equilibrium (CE) solution in this work. In contrast to the Nash equilibrium (NE), a CE solution is guaranteed to be obtained in polynomial time and is better suited to the design of the distributed optimization algorithm. The definition of CE is given below and can be interpreted as the joint probability distribution *q* of strategy profile selection to be a CE if no player *i* can choose a strategy $s_{m}^{\prime }$ instead of *s*_*m*_, resulting in higher payoff.

**Definition 1**: A correlated strategy *q* ∈ Δ(*S*) is a CE of Г if and only if for all *m* ∈ *M* and *(s*_*m*_, *s*_*-m*_ *)* ∈ *S*,

$$\sum_{s_{-m}\in S_{-m}}q(s_{m,}\;s_{-m})u(s_{m,}\;s_{-m})\geq \sum_{s_{-m}\in Sm}q(s_{m,}\;s_{-m})u(s_{m,}^{\prime }\;s_{-m}),$$ (9)

for all *ś*_*-m*_ ∈ *S*_*m*_.

## Proposed Algorithm

### Algorithm Description

**Fig 1:**
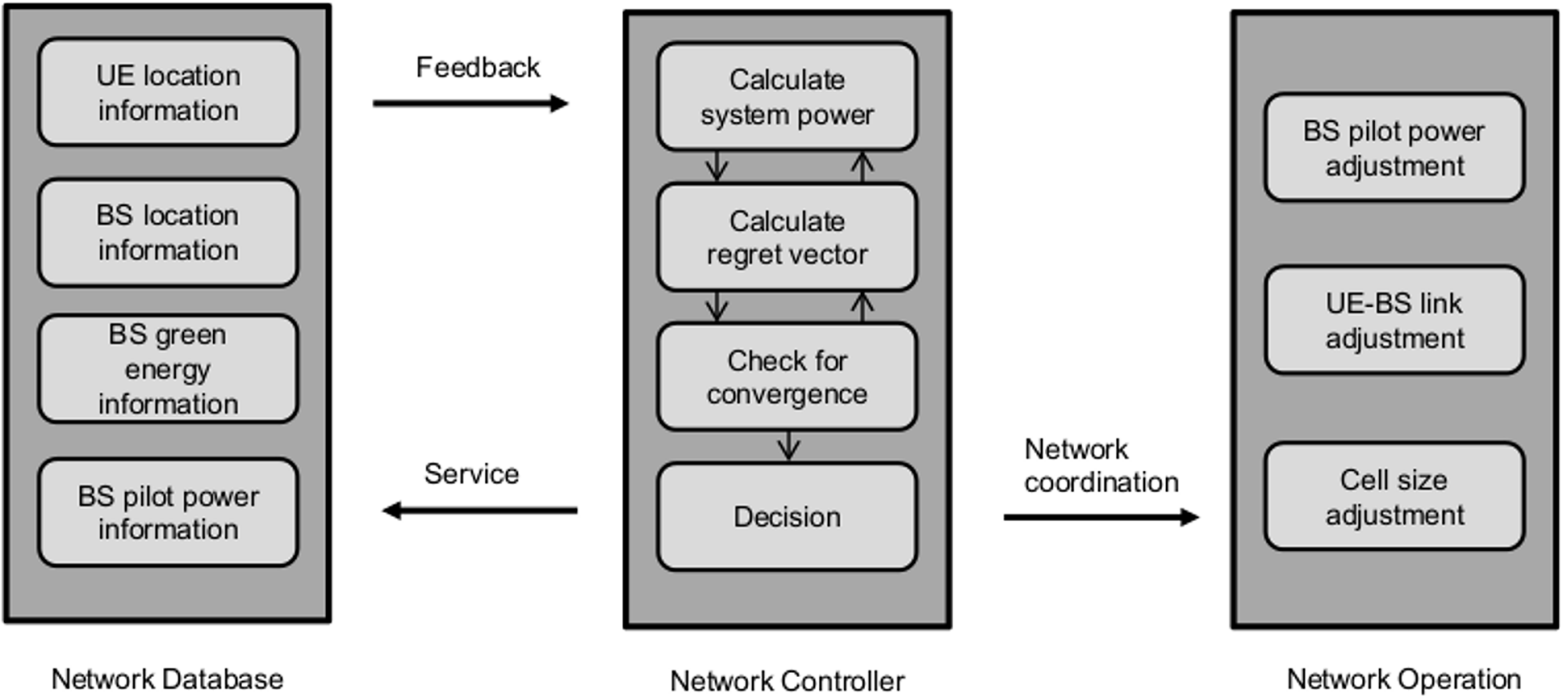
Framework of the proposed system.

**Fig 2:**
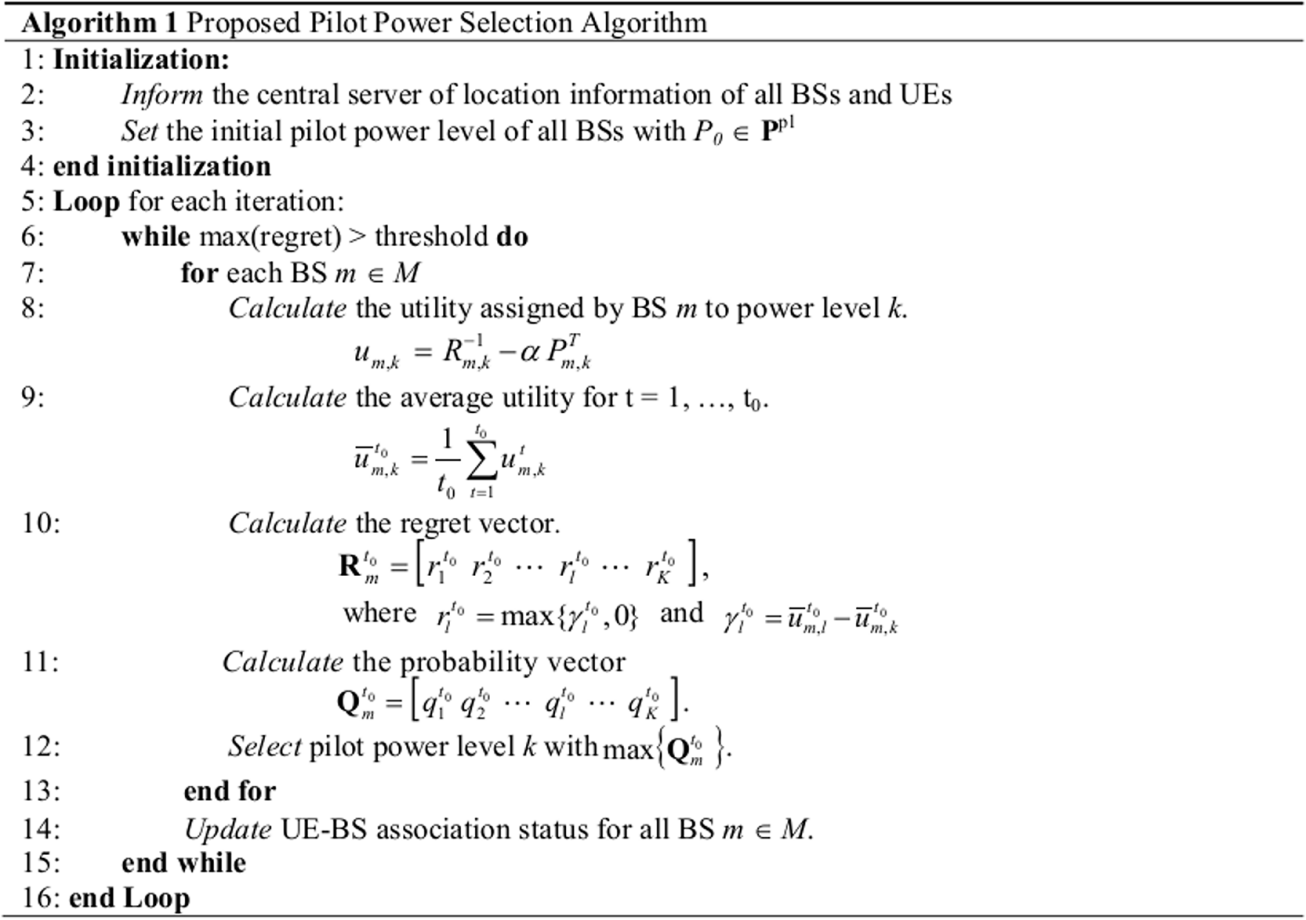
Algorithm 1: Proposed pilot power selection algorithm.

We propose a distributed algorithm to solve the pilot power selection game problem based on the regret matching procedure to achieve maximum green energy utilization and minimum total energy consumption. The regret matching procedure is an important strategic learning method, where the players adjust their strategies based on the “regrets” for not having chosen other actions [18]. In the proposed scheme, the regret for BS *m* is defined to be the difference between the total payoff due to the pilot power selection up to now and the total payoff that could have been achieved if different pilot power levels were selected. Fig 1 shows the three basic modules of the proposed system: Network Database, Network Controller, and Network Operation. The Network Database initializes the user equipment (UE) location information, base station (BS) location information, BS green energy information, and BS pilot power information, which are given to the Network Controller. Furthermore, the initial information in the Network Database is continuously updated according to the adjustments made in Network Operation. The Network Controller selects the optimum pilot power from the candidate pilot power set based on the regret matching procedure. Using the information given by the Network Database, the system power consumption and the utility for all BSs are calculated in the Network Controller. Furthermore, based on the past utilities, regrets for BS *n* for not choosing pilot power level *p*_*k*_ are calculated. Then, a decision is made if the convergence requirement is satisfied. If the convergence requirement is not satisfied, then the regret matching procedure is repeated. Based on the decision made in the Network Controller, cell size adjustments for all the BSs are performed, and the adjustment information is passed to the Network Database. Fig 2 shows the details of the proposed algorithm in pseudocode format. As seen in the figure, the algorithm has two main iterative loops, where the outer loop represents the convergence iteration and the inner loop represents the regret matching procedure for each BS.

### Convergence Analysis

**Fig 3:**
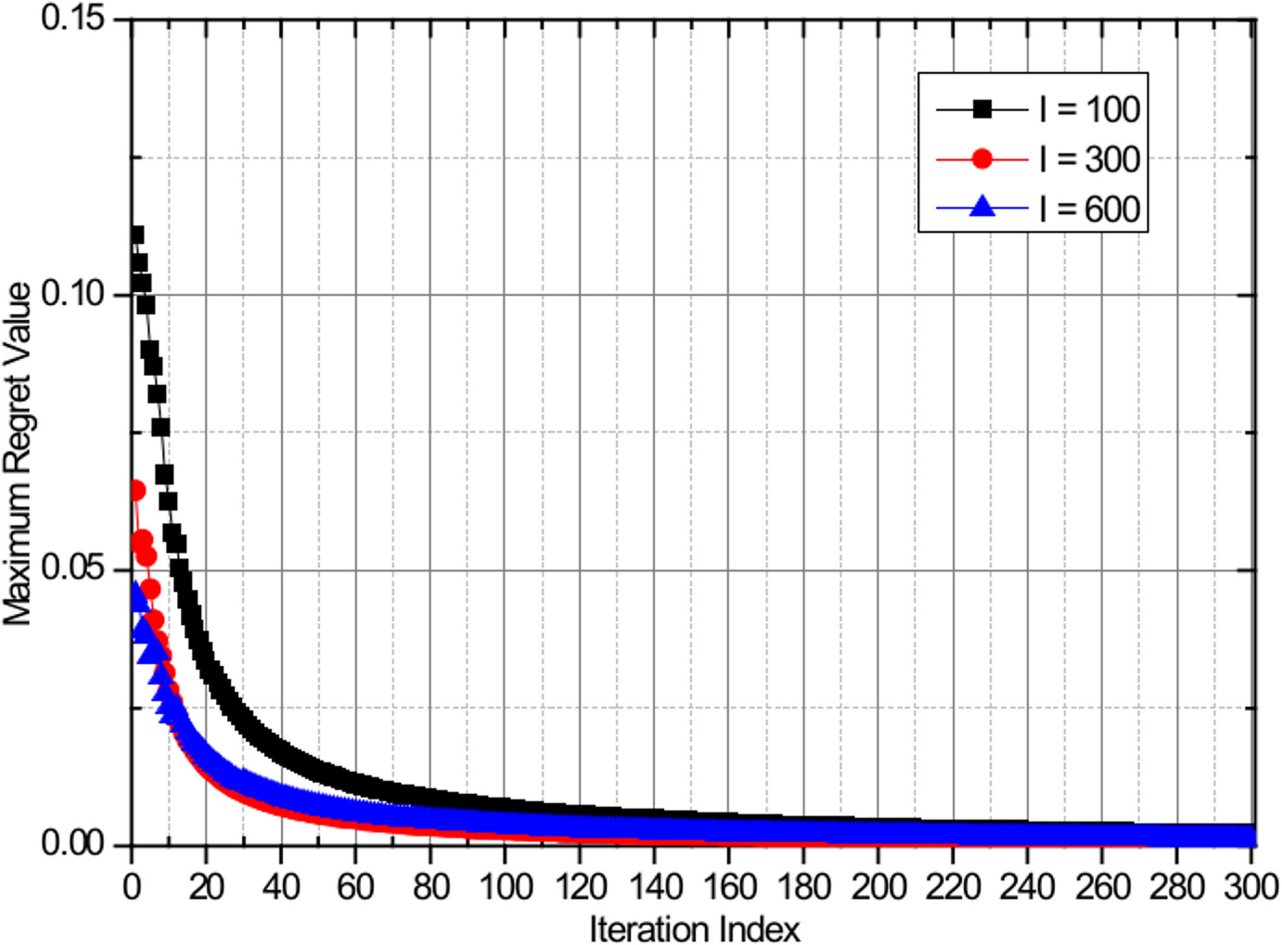
Evolution of the maximum regret value of the worst player with *I* = 100, *I* = 300, and *I* = 600 with uniform user distribution.

**Fig 4:**
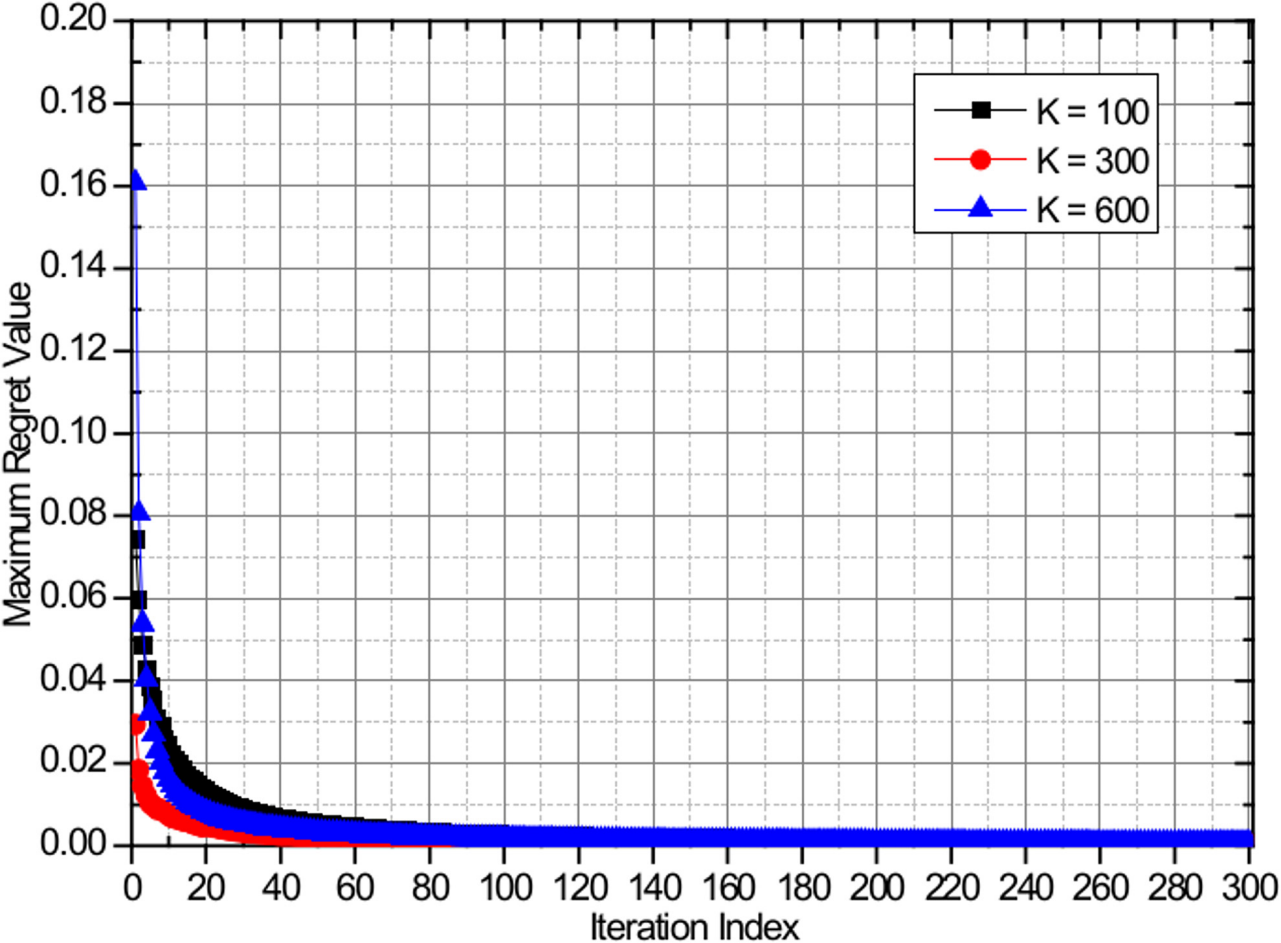
Evolution of the maximum regret value of the worst player with *I* = 100, *I* = 300, and *I* = 600 with non-uniform user distribution.

Hart and Mas-Collel proved in [18] that a necessary and sufficient condition for the empirical distribution to converge to the set of correlated equilibria is that all regrets converge to zero when the game is played according to the regret matching procedure by applying the Blackwell's approachability theorem. Because the proposed algorithm follows the regret matching procedure, the convergence to the set of coarse CE is guaranteed. The CE convergence of the proposed algorithm is evaluated by analyzing the evolution of the maximum regret value of the worst player. Fig 3 shows the evolution of the maximum regret values based on the proposed algorithm, with the maximum number of users equal to *I* = 100, *I* = 300, and *I* = 600 with uniform user distribution. Fig 4 shows the evolution of the maximum regret values based on the proposed algorithm, with the maximum number of users equal to *I* = 100, *I* = 300, and *I* = 600 with non-uniform user distribution. Regardless of the number of users and user distribution patterns, all of the regret values converge to zero in a few iterations, thus confirming that the proposed algorithm converges to the CE.

### Comparative Analysis

ICE was designed with the goal of maximizing the green energy usage by controlling the pilot power or resizing the BS. However, due to the concentration only on green energy usage optimization, there will be cases with sharp increases in the inter-distance between the UE and BS and the number of users. Therefore, the network will experience overall increase in the system energy consumption. As for the proposed method, the goal is to achieve an optimum balance between maximum green energy utilization and minimum system energy consumption. This goal is achieved by using a utility function, proposed in this paper, which is based on two important parameters: the inverse of EDR, which represents the cost of green energy efficiency, and the system power consumption. Furthermore, the proposed method is based on a regret matching procedure that guarantees optimum performance with convergence in a distributed and competitive multiplayer environment and simple implementations. However, ICE is a centralized algorithm that requires a centralized coordinator with heavy overhead between the central coordinator and all of the BSs. The SSF has the lowest complexity without any iterative adjustments, but it results in poor green energy performance due to the simple association algorithm without regard to the green energy status in the network.

## Performance Evaluation

### Simulation Environment

**Fig 5:**
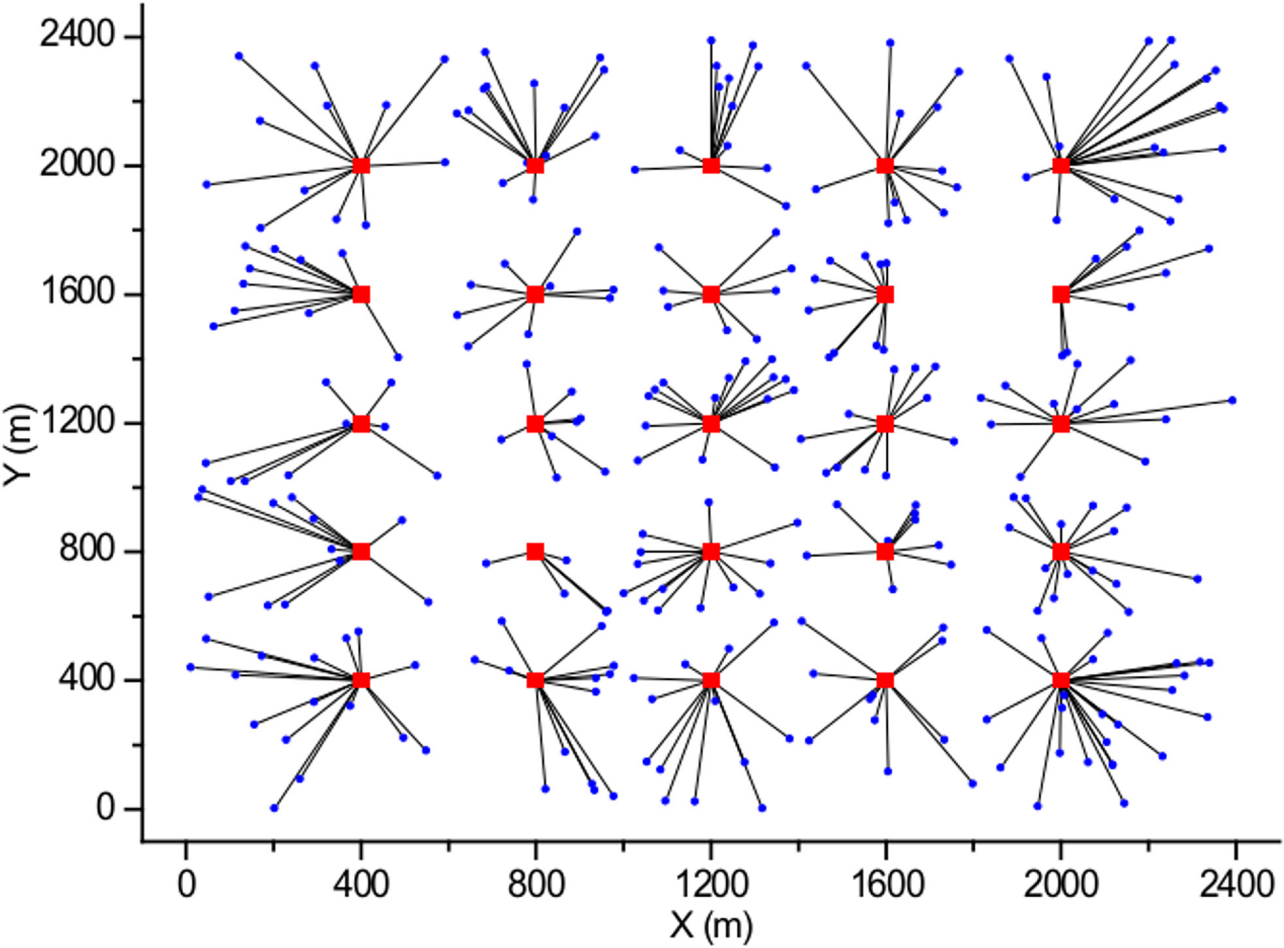
Cellular network model with SSF based BS association with a uniform user distribution.

In this section, the simulation environment for evaluation of the proposed pilot power selection algorithm based on the green energy efficiency and the system power consumption is described. In the simulations, the number of BSs is set to *M* = 25 with inter-distance of 400 m. The users are uniformly distributed in a 2400 m × 2400 m area where all the BSs are located, as shown in Fig 5. The BSs operate with the maximum transmit power of 1000 mW, and we assume that the number of pilot power levels is equal to *K* = 10 with a maximum pilot power of 50 mW. The system carrier frequency *f*_*c*_ is equal to 2 GHz, and the Gaussian noise power σ^2^ is 10^−9^ mW. Furthermore, in the proposed algorithm, the weight factor α is given the value of 10^−5^, and the number of iterations is set to 100 in all simulations. We also assume that the users do not move during the cell coverage area adjustment interval τ seconds. For comparison, the simulation results for the strongest-signal-first (SSF) scheme, which always associates a user to the BS with the strongest signal strength, as shown in Fig 5, were obtained in addition to those of ICE and the proposed scheme.

### Simulation Results

**Fig 6:**
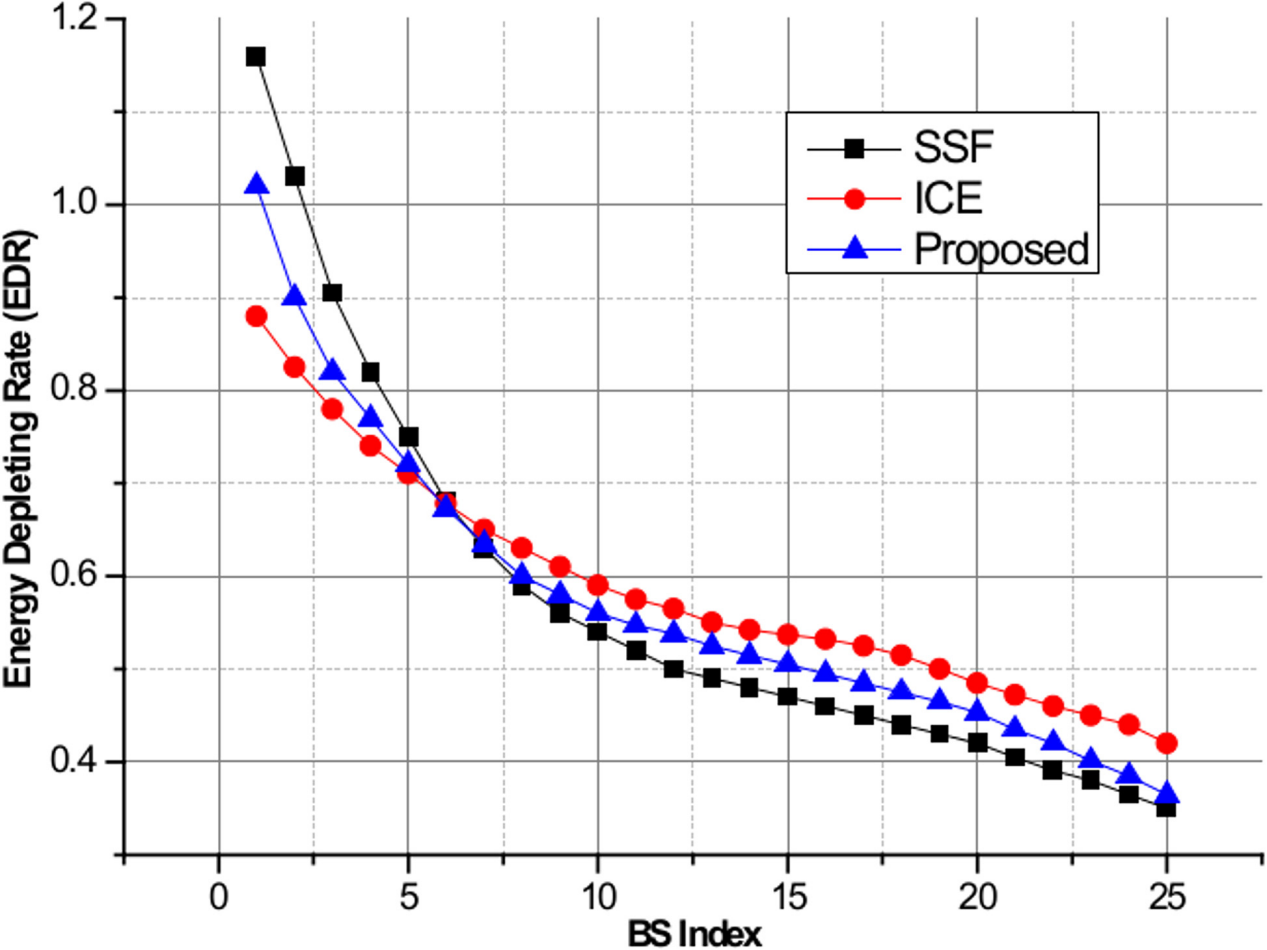
Energy depleting rate for SSF, ICE, and the proposed method.

**Fig 7:**
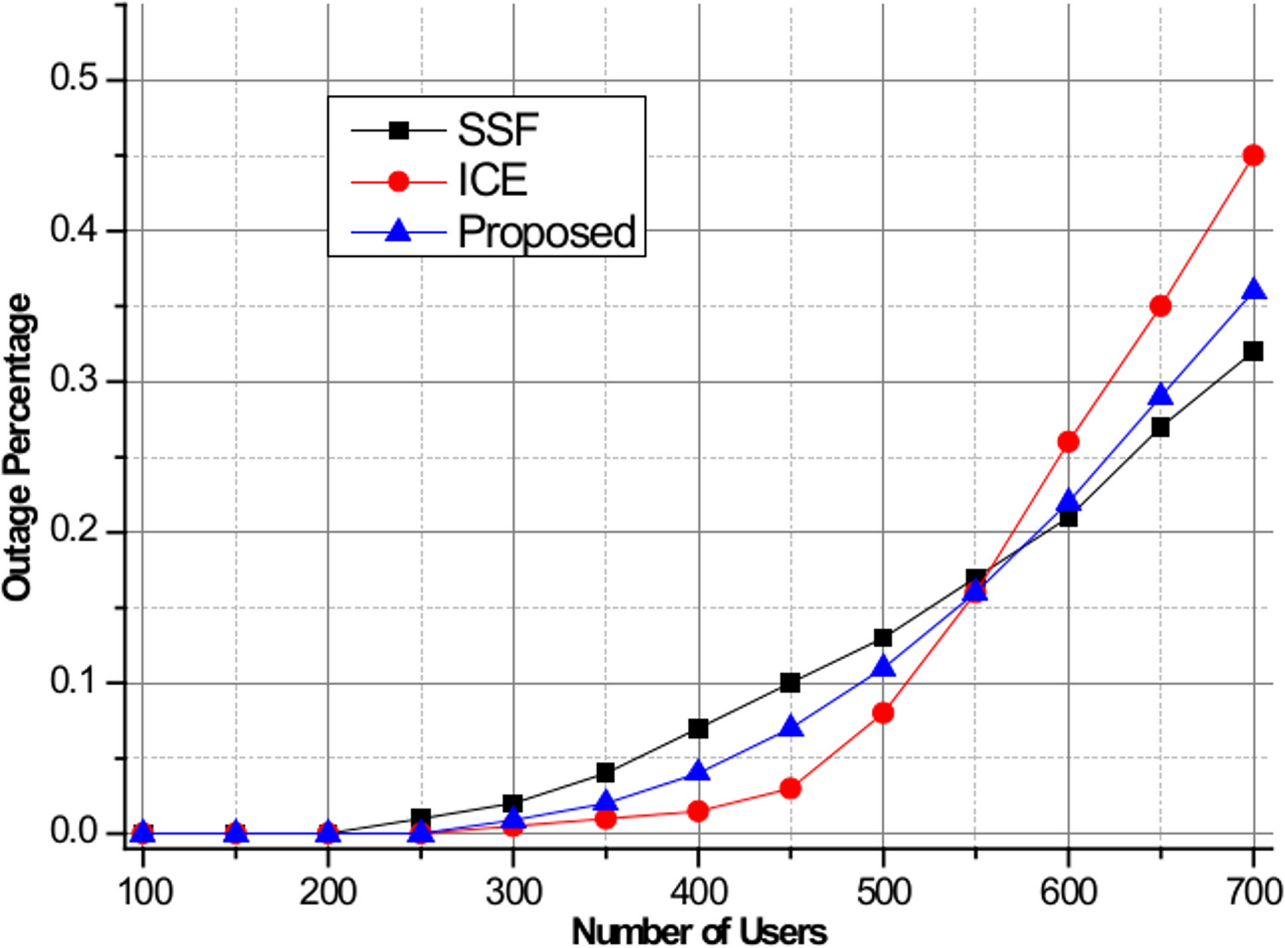
User outage percentage for SSF, ICE, and the proposed method.

**Fig 8:**
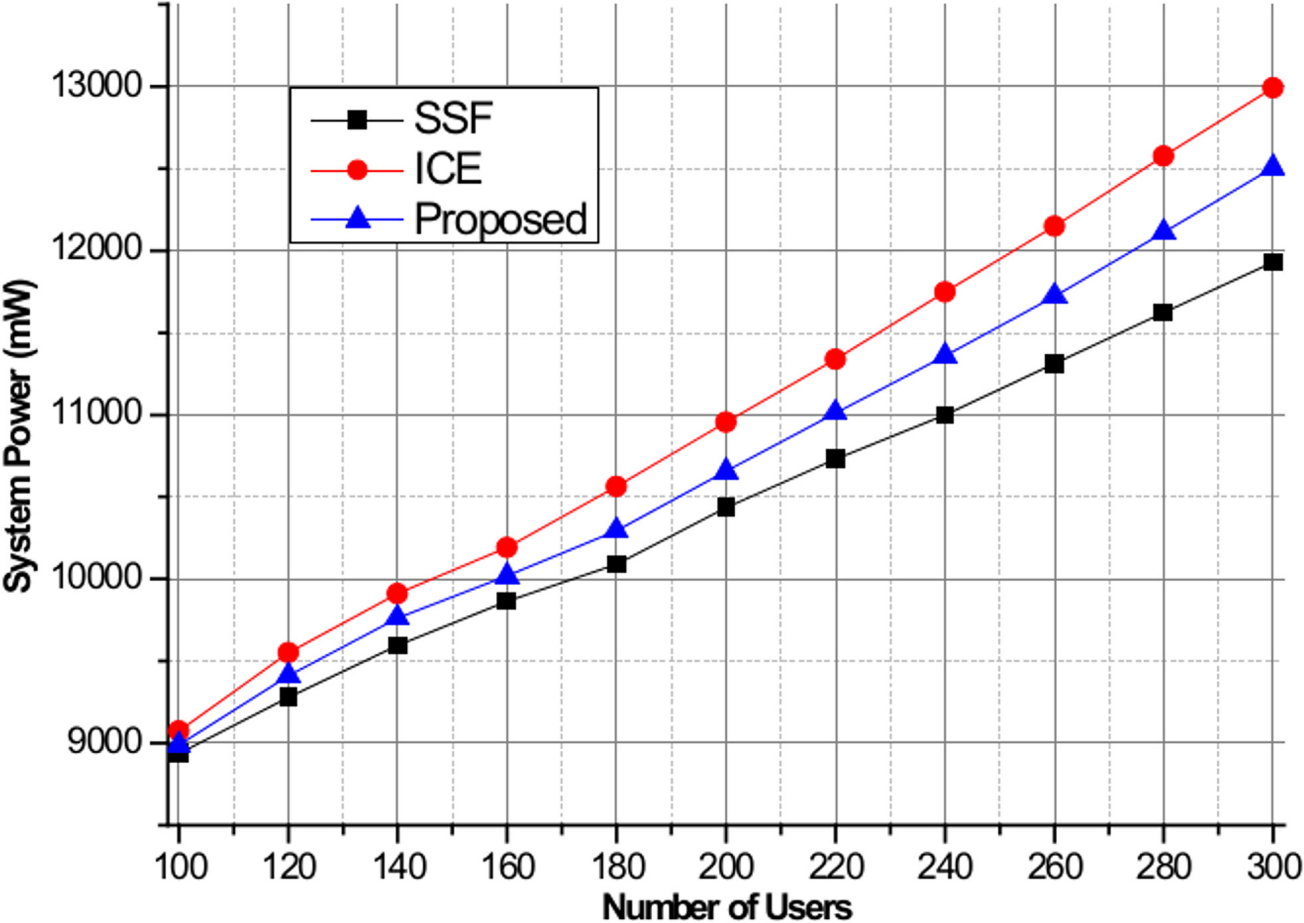
System power consumption for SSF, ICE, and the proposed method.

Fig 6 shows the maximum EDR for SSF, ICE, and the proposed scheme. We assume that all BSs have the same amount of green energy allocation of 800 mW. From the figure, we can observe that ICE successfully minimizes the maximum EDR values and balances the green energy consumption among the BSs compared to other schemes. Due to the lack of a green energy utilization optimization process, large EDR values are observed in SSF. Note that the proposed scheme experiences small EDR performance loss compared to the ICE scheme for BS indexes of 1–5, but balanced green energy utilization performance is achieved. Fig 7 shows the user outage percentage, defined as the percentage of users who are not served by green energy, for SSF, ICE, and the proposed algorithm. If the allocated green energy is not sufficient to satisfy the required power consumption, then the users in the BS are considered to be in the outage state. As seen in the figure, ICE and the proposed scheme achieve lower user outage compared to SSF when the number of users is less than 550, above which SSF achieves better performance due to insufficient green energy allocation to satisfy all users in the system. Finally, Fig 8 shows the system power consumption of SSF, ICE, and the proposed algorithm. In this simulation, the green energy allocation is randomly distributed with a uniform distribution in the range of [350, 800] mW. As the number of users increases, a significant degradation in the system power consumption performance for ICE is observed compared to the proposed algorithm. This is attributed to the design objective of ICE of minimizing the EDR metric at the expense of system power consumption. In contrast, the proposed scheme shows significant reduction in the system power consumption based on the pilot power selection game optimization, which includes the cost of the system power consumption. SSF achieves the lowest system power consumption due to the simple nearest BS selection mechanism without consideration of the green energy utilization optimization.

## Conclusions

In this paper, the problem of pilot power selection in green cellular networks was formulated as a mixed strategic game to achieve an increase in green energy utilization and a reduction in system power consumption. Furthermore, we proposed a simple distributed algorithm to optimize the green energy utilization and system power consumption of green cellular networks. The proposed scheme employs the regret matching procedure to solve the green cellular network optimization problem that is formulated as a strategic game. The simulation results have shown that the proposed scheme achieves comparable green energy efficiency to the latest green optimization technology reported in the literature and significantly improves energy consumption reduction.
